# Obesity as a Risk Factor for Severe Illness From COVID-19 in the Pediatric Population

**DOI:** 10.7759/cureus.14825

**Published:** 2021-05-03

**Authors:** Ankit Agarwal, Farida Karim, Adriana Fernandez Bowman, Callah R Antonetti

**Affiliations:** 1 Pediatrics, Ascension Sacred Heart, University of Florida, Pensacola, USA

**Keywords:** obesity, children, covid-19, risk, factors

## Abstract

In this current outbreak of coronavirus disease 2019 (COVID-19) caused by severe acute respiratory syndrome coronavirus 2 (SARS-CoV-2), many studies have been published to determine the spectrum of illness, risk factors, prevention, and treatment strategies. Due to relatively fewer cases among children as compared to adults, there is a paucity of clinical data available to fully understand the risk factors and disease course in the pediatric population. Our understanding is evolving with limited data showing an increased risk of severe or critical disease in children less than one year of age and those with certain underlying medical conditions. Recognition of emerging risk factors for morbidity and mortality is now paramount, to anticipate and provide appropriate clinical care specific to the pediatric population. Obesity has only recently been identified as a risk factor for severe COVID-19 disease in children. Case reports such as this are essential in understanding the pathophysiologic association, associated disease severity, and clinical outcome attributed to obesity and COVID-19 infections in children.

## Introduction

Coronavirus virus disease 2019 (COVID-19) is an infectious disease caused by severe acute respiratory syndrome coronavirus 2 (SARS‐CoV‐2). In December 2019, an outbreak of COVID‐19 occurred in Wuhan, Hubei Province, China [1]. On March 11, 2020, the World Health Organization (WHO) classified the outbreak as a pandemic. According to the WHO statistics, this disease has caused 3,012,251 deaths globally as of April 19, 2021.

As compared to the adult population, there are relatively fewer cases of COVID-19 among children [2,3]. In the United States, 2% of confirmed cases of COVID-19 were aged <18 years as of April 2, 2020 [3]. From March 1 to December 12, 2020, among the laboratory-confirmed cases of COVID-19 reported in the United States in children, adolescents, and young adults aged 0-24 years, 42.6% were aged 0-17 years and 0.3% had severe obesity defined as body mass index (BMI) of ≥40 kg/m^2 ^[4]. Illness among pediatric cases appears to be mild. The predominant signs and symptoms are similar to other viral respiratory infections including fever, cough, nasal congestion, rhinorrhea, sore throat, and shortness of breath. Gastrointestinal symptoms like diarrhea, nausea, or vomiting are less common manifestations [5]. According to data published by the Centers for Disease Control and Prevention (CDC), people of any age with certain underlying medical conditions such as cancer, chronic kidney disease, immunocompromised state from a solid organ transplant, sickle cell disease, obesity, type 2 diabetes mellitus, serious heart conditions are at increased risk of severe illness from COVID‐19. However, at the onset of the pandemic, obesity was not a commonly recognized risk factor in the pediatric population. While it is now specified as a risk factor for COVID-19 infection in children, the associated severity of disease and clinical course is not yet well established.

This work was previously presented as a poster at the 2020 Southern Regional Assembly and the abstract has been published in the Southern Medical Journal (2020, 113:661-821).

## Case presentation

We present a report of two cases of obese adolescent female patients with confirmed COVID-19 admitted to our hospital requiring intensive care unit (ICU) level care.

Case 1

**Figure 1:**
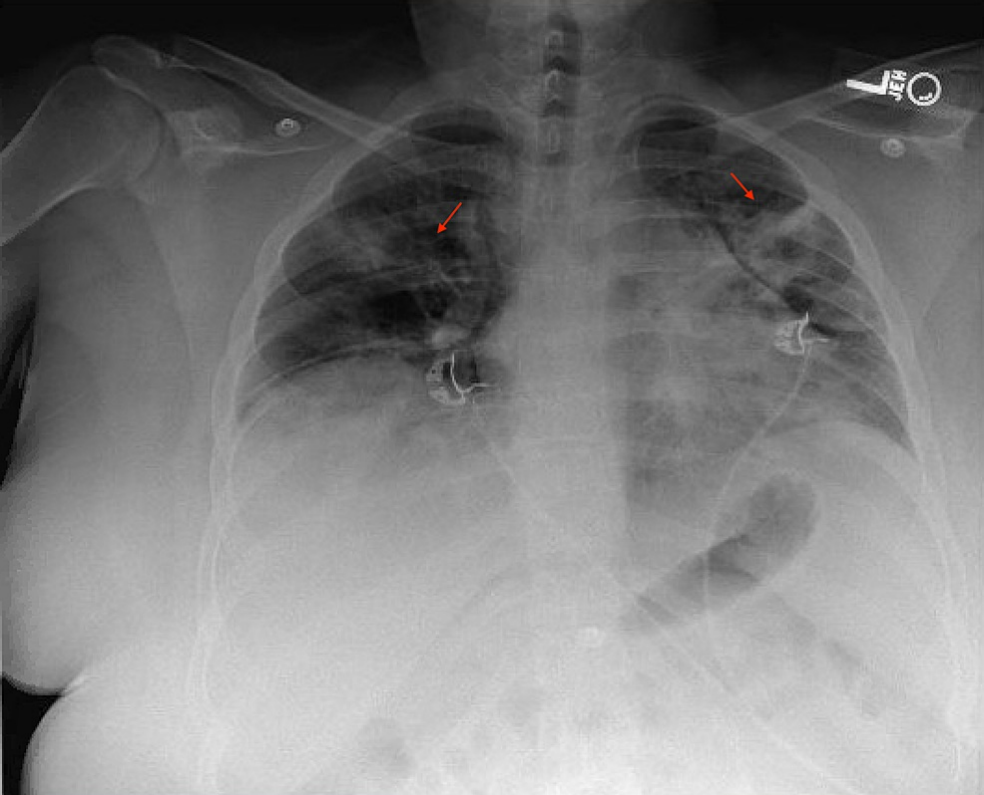
Chest radiograph showing low lung volumes with acute inflammatory infiltrates in the upper lobes accompanied by subsegmental atelectasis.

A 16-year-old white female with a BMI of 47.7 kg/m^2^, presented with a seven-day history of fever, vomiting, diarrhea, worsening cough, and shortness of breath (SOB). In the ED, she was alert with no increased work of breathing. She had episodes of pronounced hypoxemia induced by activity with associated oxygen saturations of less than 80%. On physical examination, her lungs were clear to auscultation without any sign of respiratory distress. She complained of mild abdominal tenderness on palpation. She tested positive for COVID-19 by molecular testing of the nasopharyngeal specimen. Chest radiograph (CXR) showed low lung volumes with acute inflammatory infiltrates in the upper lobes accompanied by subsegmental atelectasis (Figure 1).

She was started on 3 L/min of supplemental oxygen delivered by nasal cannula which resolved her hypoxemia. The patient developed progressively worsening hypoxemia and respiratory distress requiring escalation of oxygen support to high flow respiratory support by high-velocity nasal insufflation (HVNI) with 100% fraction of inspired oxygen (FiO2). The patient was transferred to the pediatric ICU for further management on day 2 of hospitalization. She was evaluated for COVID-19 associated multisystem inflammatory syndrome in children (MIS-C) and had laboratory evidence of inflammation with the following laboratory abnormalities: hypoalbuminemia, erythrocyte sedimentation rate (ESR) of 41 mm/hr (reference range 0-20 mm/hr), C-reactive protein (CRP) of 6.87 mg/dL (reference range <=0.90 mg/dL), fibrinogen of 445 mg/dL (reference range 200-400 mg/dL), procalcitonin of 0.27 ng/mL (reference range 0-0.10 ng/mL), D-dimer of 1.59 FEU mcg/mL (reference range <=0.49 FEU mcg/mL), ferritin of 889 ng/mL (reference range 4.6-204 ng/mL), lactic acid dehydrogenase (LDH) of 348 Units/L (reference range 25-220 Units/L), total creatine kinase (CK) of 408 Intl Units/L (reference range 29-168 Intl Units/L) and elevated prothrombin and partial thromboplastin times (PT and PTT). Echocardiogram was normal. Blood and urine cultures were negative. Due to continued hypoxemia, respiratory support was escalated to bi-level positive airway pressure. She was then intubated and placed on mechanical ventilation on day four of hospitalization. Serial CXR showed progressive worsening patchy confluent infiltrates throughout the bilateral upper and lower lung zones. She was treated with remdesivir (200 mg intravenously on day 1 followed by 100 mg daily for a total of five days), convalescent COVID-19 antibody serum, dexamethasone, intravenous immunoglobulin, and prophylactic anticoagulation therapy. As her respiratory status gradually improved she was extubated after nine days and was discharged from the hospital eight days post-extubation.

Case 2

**Figure 2:**
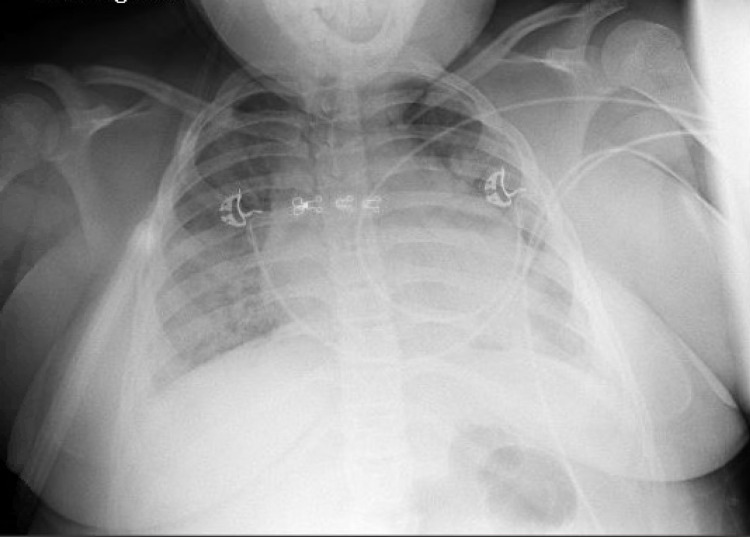
Chest radiograph showing diffuse bilateral interstitial opacities.

A 15-year-old white female with a history of poorly controlled asthma, untreated hypothyroidism, and BMI of 44.16 kg/m^2^ presented to the hospital with a five-day history of fever, congestion, cough, SOB, and headache. In the ED, she was alert and had mild respiratory distress with oxygen saturation of 86% on room air. On physical examination, she had decreased breath sounds in the bilateral lower lung lobes and crackles over the posterior lung fields. She was noted to have intermittently worsening hypoxemia with desaturations to as low as 80% with ambulation or on lying supine. Positional hypoxemia improved with change in position to lateral or prone. She tested positive for COVID-19 by molecular testing of the nasopharyngeal specimen. CXR showed diffuse bilateral interstitial opacities (Figure 2).

She was evaluated for COVID-19 associated MIS-C but did not meet the laboratory criteria. Complete blood count, comprehensive metabolic profile, PT/PTT, fibrinogen, D-dimer, and ferritin levels were all normal. CRP and ESR were elevated to 3.65 mg/dL (reference range <=0.90 mg/dL) and 54 mm/hr (reference range 0-20 mm/hr), respectively. She had an initial elevated level of total CK to 2459 Intl Units/L (reference range 29-168 Intl Units/L). Thyroid-stimulating hormone was elevated to 55.31 mcIU/mL (reference range 0.470-3.410 mcIU/mL) and the free T4 level was less than 0.40 ng/dL (reference range 0.58-1.64 ng/dL). She was started on 2 L/min of supplemental oxygen by nasal cannula with associated oxygen saturation of 92%. Over the next eight hours, she developed worsening hypoxemia with associated dyspnea, most notably during and following ambulation, and was transitioned to high flow respiratory support by HVNI with 50% FiO2. During the next 12 hours, her hypoxemia worsened, and she was transferred to the pediatric ICU and was continued on high flow respiratory support at 25 L/min with 70% FiO2 by HVNI. Her oxygen saturation improved to more than 92% but she continued to have intermittent positional and exertional hypoxemia. She was treated with albuterol, dexamethasone, prophylactic anticoagulation therapy, and thyroxine. On day 5 of hospitalization, she began showing clinical improvement and her oxygen support was weaned over the next 48 hours. She was transitioned to room air and was discharged home after seven days of hospitalization.

## Discussion

Obesity, even without additional chronic conditions, has been demonstrated to increase the risk of influenza-related complications, the risk of developing the severe disease due to respiratory viral infections, and the likelihood of hospitalization [6,7]. Similarly, adult data has shown a strong association between obesity and worse clinical outcomes in COVID-19 disease, even in the absence of any other comorbidities. In a study, obesity (BMI >30 kg/m^2^) and severe obesity (BMI >35 kg/m^2^) were present in 47.6% and 28.2% of COVID-19 cases in adults, respectively. It was found that the need for invasive mechanical ventilation was associated with severe obesity and was independent of age, diabetes, and hypertension [8].

Obesity leads to persistent immune dysregulation and is associated with increased susceptibility to infections that lead to sepsis and death [9]. It has been postulated that chronic inflammation-hypercytokinemia, endothelial dysfunction, cardiac abnormalities, and pro-thrombotic environment are the possible mechanisms through which obesity leads to worse COVID-19 outcomes [10]. The dysfunctional hypertrophic adipocytes in obesity produce an excessive amount of cytokines leading to the increased recruitment of macrophages which, in turn, produce high amounts of pro-inflammatory molecules. A published study looked at the immune responses of 54 COVID-19 patients, 28 of whom had severe respiratory failure (SRF). It was observed that all the patients with pneumonia caused by SARS-CoV-2 who developed SRF displayed hyper-inflammatory responses with features of either immune dysregulation or macrophage activation syndrome, both of which are characterized by pro-inflammatory cytokines. Over-production of pro-inflammatory cytokines by monocytes and dysregulation of lymphocytes, characterized by CD4 lymphopenia and subsequently B cell lymphopenia, are the two key features of this immune dysregulation [11]. A case series of three patients suggested that SARS-CoV-2 infection promotes the induction of endotheliitis in several organs as a direct consequence of viral involvement and the host inflammatory response; as shown by the presence of viral elements within endothelial cells and an accumulation of inflammatory cells, with evidence of endothelial and inflammatory cell death [12].

Acute cardiac injury is highly prevalent in patients with COVID-19 and is associated with worse clinical outcomes and obesity is an established risk factor for cardiovascular disease. Obesity leads to vascular abnormalities via various mechanisms and the clinical presentation might be worse in obese patients with preexisting endothelial dysfunction. Moderate to severe cases of obesity may lead to left ventricular dilation, left ventricular diastolic or systolic dysfunction, or left ventricular hypertrophy [13]. Obesity is also associated with activation of the renin-angiotensin-aldosterone system, which leads to increased levels of angiotensin II, with direct effects on the myocardium.

Based on the data collected during the March 1 to July 25, 2020, by the COVID-19-Associated Hospitalization Surveillance Network (COVID-NET), a population-based surveillance system that collects data on laboratory-confirmed COVID-19-associated hospitalizations in 14 states, 38.5% of 576 children with information on underlying medical conditions, 42.3% had one or more underlying conditions. Obesity (BMI ≥30 kg/m^2^ in a child aged ≥2 years; not evaluated for children aged <2 years) was the most prevalent condition (37.8%), followed by chronic lung disease, and prematurity (gestational age <37 weeks at birth, collected only for children aged <2 years) [14,15]. This study and other studies of hospitalized children with COVID-19 found that obesity was the most prevalent underlying medical condition [16,17].

## Conclusions

With increasing pediatric infections due to COVID-19, risk factors for disease severity are becoming evident with obesity prevailing as a major risk for the pediatric population. Childhood obesity affects almost one in every five children in the United States, and the high prevalence of obesity among children in the United States raises the risks of severe disease. Understanding the underlying pathophysiologic association between obesity and severe SARS-CoV-2 infection is important to identify possible clinical interventions and preventive strategies to reduce the risk of hospitalization and mortality.
